# Dual-Arm Visuo-Haptic Optical Tweezers for Bimanual Cooperative Micromanipulation of Nonspherical Objects

**DOI:** 10.3390/mi13111830

**Published:** 2022-10-26

**Authors:** Yoshio Tanaka, Ken’ichi Fujimoto

**Affiliations:** Faculty of Engineering and Design, Kagawa University, 2217-20 Hayashi-cho, Takamatsu 761-0396, Japan

**Keywords:** optical tweezers, cooperative micromanipulation, visuo-haptic sensing, dexterous handling

## Abstract

Cooperative manipulation through dual-arm robots is widely implemented to perform precise and dexterous tasks to ensure automation; however, the implementation of cooperative micromanipulation through dual-arm optical tweezers is relatively rare in biomedical laboratories. To enable the bimanual and dexterous cooperative handling of a nonspherical object in microscopic workspaces, we present a dual-arm visuo-haptic optical tweezer system with two trapped microspheres, which are commercially available end-effectors, to realize indirect micromanipulation. By combining the precise correction technique of distortions in scanning optical tweezers and computer vision techniques, our dual-arm system allows a user to perceive the real contact forces during the cooperative manipulation of an object. The system enhances the dexterity of bimanual micromanipulation by employing the real-time representation of the forces and their directions. As a proof of concept, we demonstrate the cooperative indirect micromanipulation of single nonspherical objects, specifically, a glass fragment and a large diatom. Moreover, the precise correction method of the scanning optical tweezers is described. The unique capabilities offered by the proposed dual-arm visuo-haptic system can facilitate research on biomedical materials and single-cells under an optical microscope.

## 1. Introduction

In biomedical studies pertaining to cell exploration, cell surgery, and cell-to-cell interaction, the precise and complex manipulation of single cells, for instance, cell isolation (translation) and three-dimensional (3D) orientation (rotation), is an essential but delicate and time-consuming task [1]. Consequently, this task can only be performed by experienced operators. Optical tweezers (the development of which received the 2018 Nobel Prize in Physics) have emerged as popular tools to handle single biological samples, and their use has been successfully demonstrated in a wide range of biomedical experiments [2,3,4]. In these experiments, the optical tweezers were directly used to manipulate the object of interest. Although the advanced direct manipulation methods based on the use of computer vision techniques and optical multiple-force clamps are valuable for biomedical studies [5,6,7], these techniques involve two key limitations: the samples may undergo photothermal damage because of exposure to a high-power laser beam, and the quality of the trap depends considerably on the shape and refraction index of the samples. To ensure the reliable handling of various biomedical samples, the indirect micromanipulation method, which represents an alternative technique of using optical tweezers, has been developed, in which optically trapped microbeads [8,9] or microstructures [10,11,12] are used as end-effectors. In the indirect micromanipulation method, the microbeads can also function as tactile or force sensors [13] because the microsphere is trapped in the center by the optical tweezers when no external forces act on the sphere. This technique, which associates tactile/force sensor functions and micromanipulation with optical tweezers, is called haptic optical tweezers and allows improved dexterity of micromanipulation. In the comprehensive review paper for haptic optical tweezers [14], the concept, design process, specification, and significant potential of optical tweezers were reviewed, focusing on haptic teleoperation. However, to the best of our knowledge, the realization of real-time multiple tactile/force sensing and its display through the use of optically trapped multiple microbeads as end-effectors (namely, dual-arm visuo-haptic optical tweezers) has not been reported yet, except in one paper [15], where the touching of cells was demonstrated by using holographic optical tweezers.

On the other hand, in general, the use of multiple tactile/force sensors and their control is essential to perform grasping or stable cooperative manipulation of a single object because multiple forces affect an object at different contact points, resulting in a stable position where the posture of the object is determined. Multi-arm/finger robot systems have become the prevalent platform for performing precise and dexterous tasks in medical surgery [16,17] and automation [18,19,20]. However, in biomedical laboratories, the implementation of cooperative micromanipulation with multiple end-effectors or microrobots [12] is relatively rare, except in the case of intracytoplasmic sperm injection [21].

Therefore, to enable the cooperative handling of a nonspherical object in microscopic workspaces, in this paper, we present dual-arm visuo-haptic optical tweezers with two optically trapped microspheres, which are commercially available end-effectors to realize indirect micromanipulation. Through the combination of the precise correction of distortions in scanning optical tweezers and computer vision techniques to detect multiple microspheres, the system enables an operator to perceive the real contact forces during the cooperative manipulation of an object. In this manner, the system helps enhance the dexterity of bimanual micromanipulation by employing the real-time representation of the forces and their directions. As a proof of concept, we demonstrate the cooperative indirect micromanipulation for two types of single nonspherical samples: a glass fragment and a large diatom. Moreover, we describe the precise correction method of the scanning optical tweezers.

## 2. Dual-Arm Visuo-Haptic Optical Tweezers

### 2.1. System Design and Experimental Setup

Recently, we developed a dual-arm optical tweezer system to enable precise and dexterous micromanipulation in the 3D workspace [22]. In general, dual-arm systems extend the possibilities of research in the domain of single-cell and 3D biology. However, to perform more precise and complex tasks such as cooperative manipulation, the use of two PC mice as user interface devices for bimanual 3D control is inadequate because it is necessary to realize simultaneous tactile/force sensing at multiple positions with effective control to stabilize the object handled by the multiple end-effectors [14]. In Ref. [15], the touching of red blood cells was only demonstrated in the quite limited 2D narrow workspace (10×10 μm), although the real force feedback was performed by the bilateral teleoperation of holographic optical tweezers. In this section, to realize the bimanual cooperative handling of various nonspherical objects in a wider workspace (roughly, from 50×50 μm to 100×100 μm squares), we describe the design of a dual-arm visuo-haptic optical tweezer system with two optically trapped microspheres.

**Figure 1:**
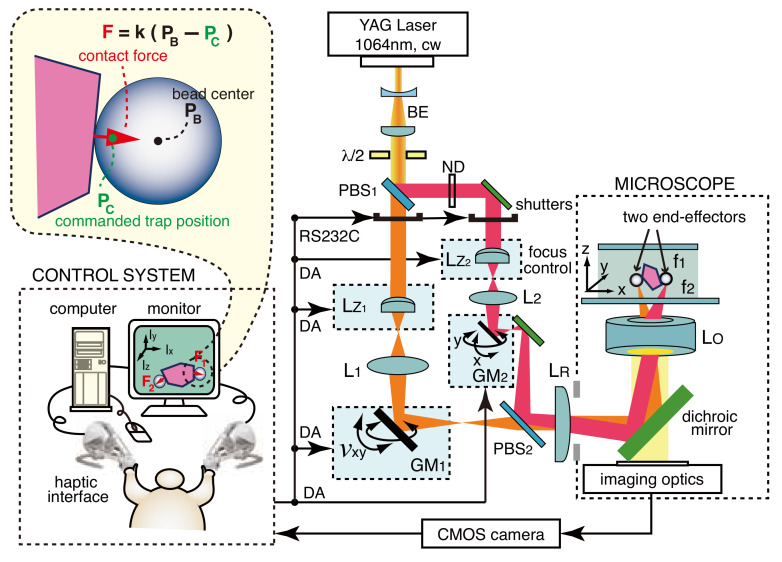
Schematic of dual-arm visuo-haptic 3D optical tweezer system that can represent the forces during indirect micromanipulation with two microspheres.

Figure 1 shows the optical and control system configurations of the dual-arm visuo-haptic 3D optical tweezers involving two haptic devices (Novint Falcon^®^s). This optical structure was linked to the inverted microscope (Olympus IX70) via its epifluorescence port. The laser source was a continuous wave (cw) YAG laser (Laser Quantum Opus 1064-5000, 1064 nm, TEM_00_, 5 W(max)), and the beam diameter was expanded to roughly 4 mm by a beam expander (BE). We used one computer (Windows7, Intel^®^ Core™ i7-4790, 3.6 GHz) as the control system. The system pertains to two-beam scanning optical tweezers, in which the two beams divided by a polarized beam splitter (PBS${}_{1}$) individually configure the true 3D optical tweezers through an electrical focus tunable lens (L${}_{Z}$: Optotune EL-10-30-NIR-LD) for *z*-coordinate steering and a two-axis scanning gimbal mirror (GM: Newport FSM-300) for *x*–*y* plane steering [23]. These two true 3D optical tweezers, in which the focal position of each trapping beam can be fully controlled in the 3D workspace, employed the 2f relay optical system under the common relay lens (L${}_{R}$: $f_{R}=200$ mm). In particular, although 4f afocal relay systems are often employed in multibeam optical tweezers involving a spatial light modulator, optical tweezers based on the 2f relay systems require fewer optical elements resulting in enhanced light transmission, fewer potential optical aberrations, and a simpler alignment process. To realize simultaneous tactile/force sensing at a specific contact position of the two end-effectors, we applied the well-established fact that a microsphere is trapped at its center (P${}_{B}$) by optical tweezers if no external forces affect the microsphere, that is, P${}_{B}$ equals the commanded focus position (P${}_{C}$) of a trapping beam; the restoring force of the optical tweezers for a microsphere is simply proportional to the distance between P${}_{B}$ and P${}_{C}$. Consequently, the contact force (*F*) is

(1) $$F=k(P_{B}-P_{C}),$$

where *k* is the spring coefficient of the optical tweezers [24]. Therefore, the accurate detection of the center of an individual bead and precise control of each beam focus corresponding to P${}_{C}$ are extremely important to represent the contact forces. The circular Hough transform algorithm pertaining to real-time image processing techniques [25,26] was applied to detect the bead centers for the real-time microscopic image, which was captured using a fast USB3 camera (Point Grey GS3-U3-41C6C-C), on which the force arrows (indicating the calculated force vectors) determined using Equation (1) were superimposed. The digital-analog (DA) signal commands were used to control the beam focus positions. The force arrows enable us to visually perceive the contact forces exerted during the cooperative manipulation of a single object with two trapped beads.

### 2.2. Correction Method for Field Distortion in a Dual-Arm System

As mentioned previously, to represent a force arrow, it is necessary for the actual focus position to exactly correspond with P${}_{C}$, since we assume that a microsphere is trapped at P${}_{B}$ by the optical tweezers under a lack of external forces. Hence, to realize visuo-haptic manipulation, the error between the actual and commanded focus positions must be compensated over the entire workspace in which the trapped bead (that is, the end-effector) can be handled by controlling the two-axis scanning mirror. In this section, we describe the precise correction method of the scanning optical tweezers.

As shown in Figure 1, the dual-arm visuo-haptic system involves two-beam scanning optical tweezers, with each beam constructing the optical tweezers through a pre-objective type scanning control system; in this system, the scanning mirror (GM) was located in front of the objective lens of the microscope (L${}_{O}$), which was an ideal F-theta focusing lens [27]. Moreover, the common relay lens (L${}_{R}$), which projects the image onto the scanning mirrors onto the back pupil of L${}_{O}$, was inserted in each scanning optical tweezer system. In general, the focus position of a laser beam in an F-theta scanning system is linear to the commanded angle of a scanning mirror. However, in the dual-arm scanning system, the focus position of each trapping beam is significantly influenced by L${}_{R}$, which leads to field distortion. Notably, the individual field distortion of the dual-arm scanning system, which is composed of tangential distortion and asymmetric radial distortion, is mainly generated by the optical path error, which occurs via the decentering, tilt, and aberration of L${}_{R}$. Consequently, the focus position of each trapping beam is not linear to the commanded angle of each two-axis scanning mirror. This nonlinearity of the commanded focus positions, caused by the field distortions, can be corrected using a compensation function. Considering the tangential and radial distortion [27,28], the compensation function of the individual two-axis scanning system can be expressed using a two-dimensional five-order polynomial function Θ(x,y):

(2) $$\begin{array}{cc} \Theta (x,y) & =a_{0}+a_{1}x+a_{2}y+a_{3}xy+a_{4}x^{2}+a_{5}y^{2}+a_{6}x^{2}y+a_{7}xy^{2}+a_{8}x^{3}+a_{9}y^{3}+a_{10}x^{5}\\ \; & \;+a_{11}y^{5}+a_{12}x^{4}y+a_{13}xy^{4}+a_{14}x^{3}y^{2}+a_{15}x^{2}y^{3}, \end{array}$$

where (x,y) denotes the specified focal position (P${}_{C}$), determined using a camera under the *x*–*y* imaging coordinates, and Θ(x,y) corresponds to the commanded DA signal (voltage) for the *x*–*y* positioning angles of the two-axis mirror; we considered two voltage values $\Theta =(v_{x},v_{y})$ for the *x*- and *y*-axes. In Equation (2), the coefficient $a_{0}$ expresses the offset pertaining to the decentering of L${}_{R}$; the coefficients $a_{1},\ldots ,a_{5}$ express the effect of the tangential distortion owing to the tilt of L${}_{R}$ to the optical axis of L${}_{O}$; and the coefficients $a_{6},\ldots ,a_{9}$ and $a_{10},\ldots ,a_{15}$ express the effect of the radial distortion caused by the tilt and aberration of L${}_{R}$, considering the effect of term $r_{2}\;\left(r^{2}=x^{2}+y^{2}\right)$ and $r^{4}$, respectively.

For *N* sets of measured data $\left(v_{x},v_{y},x,y\right)$, Equation (2) can be represented in the following matrix form:

(3) Θ=MA,

where

(4) $$\Theta =\left(\begin{array}{cc} v_{x_{1}} & v_{y_{1}}\\ v_{x_{2}} & v_{y_{2}}\\ \vdots & \vdots \\ v_{x_{N}} & v_{y_{N}} \end{array}\right)\in R^{N\times 2},$$

(5) $$M=\left(\begin{array}{ccccccccccccc} 1 & x_{1} & y_{1} & x_{1}y_{1} & x_{1}^{2} & y_{1}^{2} & x_{1}^{2}y_{1} & \cdots & y_{1}^{3} & x_{1}^{5} & \cdots & x_{1}^{3}y_{1}^{2} & x_{1}^{2}y_{1}^{3}\\ 1 & x_{2} & y_{2} & x_{2}y_{2} & x_{2}^{2} & y_{2}^{2} & x_{2}^{2}y_{2} & \cdots & y_{2}^{3} & x_{2}^{5} & \cdots & x_{2}^{3}y_{2}^{2} & x_{2}^{2}y_{2}^{3}\\ \vdots & \vdots & \vdots & \vdots & \vdots & \vdots & \vdots & \ddots & \vdots & \vdots & \ddots & \vdots & \vdots \\ 1 & x_{N} & y_{N} & x_{N}y_{N} & x_{N}^{2} & y_{N}^{2} & x_{N}^{2}y_{N} & \cdots & y_{N}^{3} & x_{N}^{5} & \cdots & x_{N}^{3}y_{N}^{2} & x_{N}^{2}y_{N}^{3} \end{array}\right)\in R^{N\times 16},$$

(6) $$A=\left(\begin{array}{cc} a_{x_{0}} & a_{y_{0}}\\ a_{x_{1}} & a_{y_{1}}\\ \vdots & \vdots \\ a_{x_{14}} & a_{y_{14}}\\ a_{x_{15}} & a_{y_{15}} \end{array}\right)\in R^{16\times 2}.$$

To determine the unknown coefficients, $a_{0},\ldots ,a_{15}$, in the compensation function presented in Equation (2), we solve the matrix equation presented in Equation (3). Using a pseudoinverse matrix $M^{†}$, we can obtain the coefficient matrix A:

(7) $$A=M^{†}\Theta ,$$

where

(8) $$M^{†}={\left(M^{⊤}M\right)}^{-1}M^{⊤},$$

in which superscripts ⊤ and −1 denote the transpose and inverse of a matrix, respectively [29]. If the rank of M is less than 16, Equation (3) has no solution. Hence, the number of the measured data sets must be N≥16. Note that the data set $\left(v_{x},v_{y},x,y\right)$ must be uniformly collected over the complete workspace, and the number N>100 is adequate to satisfy the rank M=16 and avoid the calculation of Equation (8) under the numerically ill condition.

**Figure 2:**
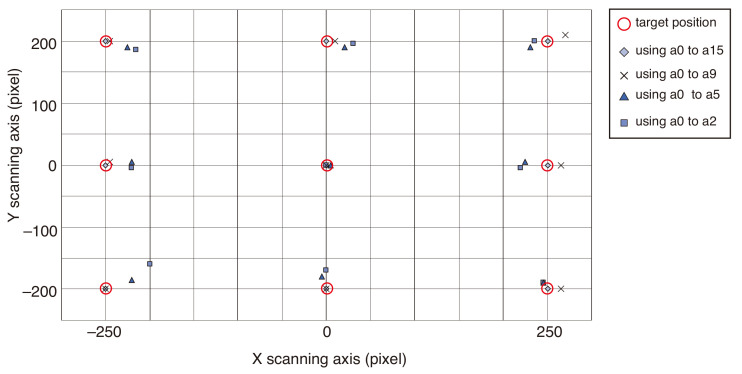
The result of the correction for distortions in the scanning optical tweezer system. The errors pertaining to the target focus positions are indicated by four symbols under a five-times-expanded scale. The number of measured data points is N=119. The 512×440 pixel imaging plane is equivalent to the 138×118 μm real workspace for ×40 objective lens, the 92×79 μm for ×60, and the 54×47 μm for ×100, respectively.

Figure 2 shows the result of the corrections for the field distortions in the proposed dual-arm scanning system. The errors in four cases involving different coefficients are indicated by different symbols (⋄, ×, etc.) under a five-times-expanded scale. The data were collected by the low-magnification objective lens (Olympus, LCPlanFL, ×40, 0.6 NA), and the number of measured data points for the calculation of Equation (7) was N=119. When coefficients $a_{0}$ to $a_{5}$ were used, only the tangential distortion was compensated. When coefficients $a_{0}$ to $a_{9}$ and $a_{0}$ to $a_{15}$ were used, the radial distortion of the relay lens was compensated as well, considering the effect of the terms $r^{2}$ and $r^{4}$, respectively. When coefficients $a_{0}$ to $a_{2}$ were used, the distortions due to the relay lens are not compensated. When all coefficients from $a_{0}$ to $a_{15}$ concerned with the field distortions were applied, the error between the actual target and commanded focus positions was compensated over the complete workspace of the imaging plane of the microscope with 512×440 pixels.

## 3. Demonstrations and Discussion

### 3.1. Bimanual Control of End-Effectors

To verify whether the abovementioned correction method can sufficiently reduce the error between the actual and commanded focus positions, we demonstrate the bimanual and simultaneous tactile/force sensing with two optically trapped microbeads as end-effectors to realize cooperative indirect micromanipulation.

**Figure 3:**
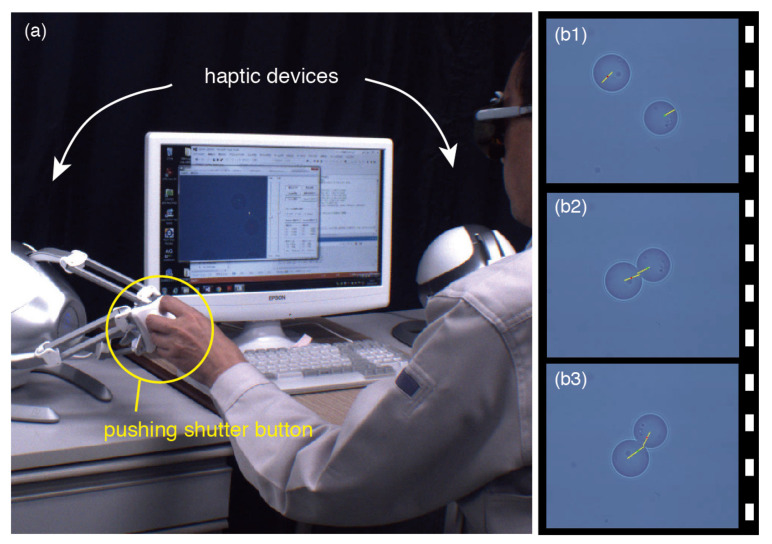
(**a**) Snapshot of the 3D position control of end-effectors (two 7.8 μm glass microbeads) through two haptic devices. (**b**) Video frame sequence of the simultaneous handling of the two microbeads. The tweezing beam positions controlled using the haptic devices are superimposed by the green and red circles. The forces generated by the contact or viscous drag are also superimposed in real time at the point of action by the yellow lines. The images are also shown in Supplementary Videos S1 and S2.

Figure 3a shows the snapshot of the 3D position control of the end-effectors (Duke Scientific, borosilicate glass microsphere, 7.8 μm) based on two haptic devices. The laser powers for trapping the individual end-effectors were adjusted to the equivalent value (50 mW) at the entrance aperture of the objective lens (Olympus, UPlanSApo, ×100, 1.40 NA, IR). As shown in Figure 3a and its Supplementary Videos, the operator, bimanually and independently, manipulated two microbeads, while visually perceiving the forces generated by their contact or the viscous drag. Figure 3b shows the video frame sequence of the simultaneous handling of two end-effectors, in which the beam focus positions, controlled by the individual haptic devices, were superimposed on the monitor of the PC as the green and red circles, which could not be observed through the fast USB camera. Furthermore, the forces generated by the contact or viscous drag were superimposed in real time at their point of action by the yellow lines, whose lengths were proportional to the forces. In Figure 3b1, the individual viscous drag caused by the surrounding material (water), which acted on each trapped microbead in the direction opposite to its movement and was proportional to the velocity of the movement, is represented by a yellow line. In Figure 3b2,b3, the reaction forces on the two microbeads in contact are displayed at the point of contact in real time. When the two beads contact each other, the two yellow lines have almost equal length and indicate the same point at which the microbeads are in contact; thus, we can verify the complete calibration through the correction method.

### 3.2. Cooperative Micromanipulation of Nonspherical Objects

To demonstrate the enhanced stability and dexterity of bimanual micromanipulation by using the visuo-haptic information, we performed simple cooperative handling tasks for nonspherical objects. The end-effectors for indirect micromanipulation were two microbeads (Duke Scientific, borosilicate glass microsphere, 7.8 μm), and the laser powers for trapping the individual end-effectors were adjusted to the equivalent value (50 mW) at the entrance aperture of the objective lens (Olympus, UPlanFLN, ×60, 1.25 NA, IR).

**Figure 4:**
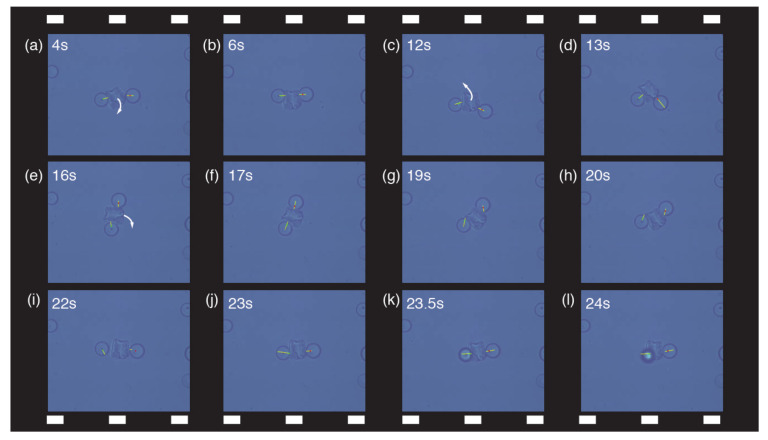
Video frame sequence of the cooperative indirect micromanipulation of a glass fragment, with the reaction forces and their directions at each contact position of the end-effectors (7.8 μm microbeads) superimposed by yellow lines in real time. In each snapshot, the movement direction of the fragment during cooperative manipulation is indicated by a white arrow. The images are also shown in Supplementary Video S3.

Figure 4 shows the video frame sequence of the bimanual and indirect cooperative micromanipulation of a single glass fragment, with the reaction forces and their directions at each contact point of the end-effectors superimposed by yellow lines in real time. In each snapshot of the video frame, the moving direction of the glass fragment during the cooperative manipulation is indicated by a white arrow, and the captured time, as obtained from the Supplementary Videos, is indicated at the left-upper corner. The visuo-haptic information represented by the yellow lines allows the intuitive perception of the individual reaction force when the microbeads are in contact with the glass fragment and the individual viscous drag when the microbeads move through water. In this manner, the real-time visuo-haptic information helps the user apply and control the suitable forces to realize the cooperative pushing/pinching of the nonspherical object during the bimanual manipulation. For example, when the operator pushed the glass fragment with the two beads that contacted on the fragment’s opposite sides, the fragment affected by the two different forces at different action points was rotated to the desired posture (Figure 4a,b,e–k); when the operator also pushed the fragment with two beads that contacted the same side, the fragment was translated to the desired position, rotating to its stable posture (Figure 4c–e). Consequently, the cooperative pushing realized through two microbeads with different forces and action points helped to rotate/translate the fragment without the bead escaping the trap, as shown in Figure 4l. In other words, by exploiting the visuo-haptic information (that is, through the yellow lines representing the current forces), 2D cooperative handling of single nonspherical object pushed/pinched by two end-effectors was robustly performed.

**Figure 5:**
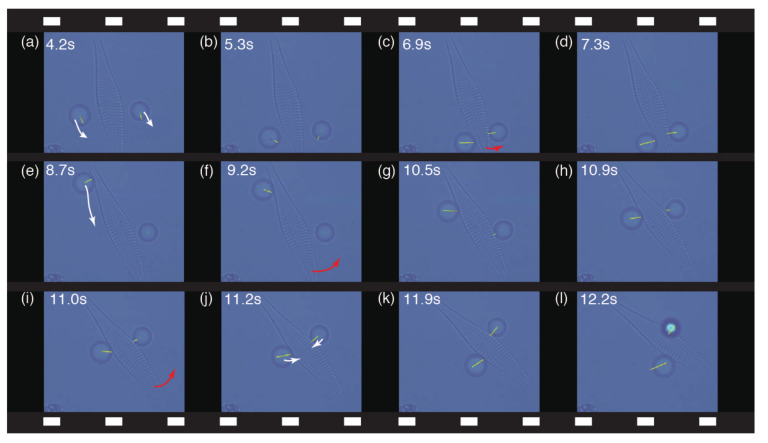
Video frame sequence of the cooperative indirect micromanipulation of a large diatom, with the exerted forces and their directions on the end-effectors (7.8 μm microbeads) displayed by yellow lines in real time. In each snapshot, the movement direction of the microbeads and the diatom during cooperative manipulation are indicated by white and red arrows, respectively. The images are also shown in Supplementary Video S4.

In another demonstration, as shown in Figure 5, a single diatom which had ellipse-like cell walls of silica—larger and heavier than the abovementioned glass fragment—was handled using the two microbeads; the forces exerted on the microbeads are displayed by yellow lines in real time. In each snapshot of the video frame, the movement direction of each microbead and that of the diatom during cooperative manipulation are indicated by white and red arrows, respectively. First, to examine the suitable forces and corresponding movement of the diatom, we interactively performed pushing/pinching motions on both sides of the diatom while monitoring the superimposed forces (Figure 5a–d). Next, the left bead was moved to contact the left-upper edge of the diatom (Figure 5e) and pushed along the left edge of the diatom from its upper to center position, as indicated by the white arrow in Figure 5e; this maneuver resulted in the diatom being gradually rotated, as indicated by the red arrows in Figure 5f,i. Finally, the right bead pushing the diatom escaped its trap (Figure 5l).

Without real-time visuo-haptic information or force feedback [13], the cooperative handling of a heavy and rigid object such as the considered diatom is a difficult task. The beads used as end-effectors often escape from their optical traps because the bead that pushes the rigid object on one side is often subjected to an unexpected and undesired large reaction force exerted by the other bead that is supporting/pushing the object on the opposite side. In this context, by exploiting the real-time representation of the reaction forces, we can perceive and control the corresponding pushing forces within a desirable range, thereby alleviating the escape problem. Thus, the proposed dual-arm system exhibits an enhanced robustness in the conduction of cooperative micromanipulation tasks.

## 4. Conclusions

We designed a dual-arm visuo-haptic optical tweezer system in which a user can perceive real forces during bimanual micromanipulation. Moreover, we presented a precise correction method for the field distortion of scanning optical tweezers in a wide workspace to represent the force vectors affecting the end-effectors during cooperative indirect micromanipulation. Although we attempted to realize the cooperative indirect micromanipulation for only two types of single samples in a limited (that is, in a 2D and not 3D) workspace because of the insufficient performance of the used PC, it was demonstrated that the system could help to realize highly robust indirect micromanipulation (that is, ensuring the continuous locking of microbeads as end-effectors in the optical tweezers) during cooperative manipulation involving visuo-haptic information. A possible application based on the straightforward implementation of 3D cell rotation by multi-fingers (namely, multiple traps) in a 3D workspace is tomographic imaging and 3D microscopy for nonspherical cells [30]. As future work, we plan to expand the performance of image processing and multiple traps from 2D to 3D traps by replacing the PC. Therefore, the proposed dual-arm system can enable bimanual and dexterous handling of various micro-objects. Furthermore, the capabilities offered by this system can facilitate novel research in the domain of biomedicine (e.g., knotting [31], bridging [32], and twisting of cell/biomaterials) as well as single-cell biology, performed under optical microscopes.

## Data Availability

Not applicable.

## Supplementary Materials

The following supporting information can be downloaded at: https://www.mdpi.com/article/10.3390/mi13111830/s1, Video S1: 3D position control of end-effectors; Video S2: Simultaneous handling of the two microbeads; Video S3: Cooperative indirect micromanipulation of a glass fragment; Video S4: Cooperative indirect micromanipulation of a large diatom.
